# Can patient-derived in vitro models improve clinical translation in critical care research when used before animal studies?

**DOI:** 10.1186/s40635-025-00814-z

**Published:** 2025-10-15

**Authors:** Alexandre Pierre, Raphael Favory, Steve Lancel, Sebastien Preau

**Affiliations:** 1 U1167 - RID-AGE - Facteurs de Risque Et Déterminants Moléculaires Des Maladies Liées Au Vieillissement, Univ. Lille, Inserm, CHU Lille, Institut Pasteur de Lille, F-59000 Lille, France; 2 Division of Intensive Care, Hôpital Roger Salengro, CHU de Lille, 59000 Lille, France

## Abstract

**Background:**

Translational failure remains a major barrier in critical illness research, with preclinical findings from animal models often failing to replicate in human trials. Hypothesis: we hypothesize that the integration of advanced in vitro models derived from human cells—particularly those from ICU patients—prior to animal studies will enhance clinical translation in critical care research.

**Main text:**

These emerging human-relevant platforms—such as organ-on-chip microfluidic systems—recapitulate key aspects of human physiology and pathology that animal models often cannot, thereby avoiding interspecies differences, capturing patient-specific variability, and enabling the study of disease phenotypes and endotypes. We propose that advanced in vitro models should be used first to gain mechanistic insights and assess efficacy in a human-relevant setting, while subsequent animal studies would then serve to evaluate systemic effects and safety before translation to patients. By leveraging such complementary strengths, an integrated in vitro–in vivo pipeline could better bridge the bench-to-bedside gap. This approach aligns with 3Rs principles by refining and reducing animal use (screening therapeutics in human models to focus subsequent animal experiments), and potentially replacing certain animal tests pending rigorous validation and regulatory acceptance. Implementation will require regulatory support, as well as training and funding to overcome technical barriers. This hypothesis is testable through analyses of past translational failures to determine whether human in vitro models could have predicted outcomes, and through prospective studies comparing drug development pipelines with and without an in vitro prescreening step to assess improvements in clinical success rates.

**Conclusions:**

By harnessing the strengths of both model systems, this two-step strategy could help bridge the translational gap in critical care, improve therapeutic development, and accelerate precision medicine in sepsis and other critical illnesses.

## Introduction

The translational gap between preclinical models and clinical outcomes in humans remains a persistent and well-recognized challenge [1]. The failures in the development of drugs to treat sepsis or ARDS underscore the limitations of relying solely on animal models to understand the complex, multi-cellular, and organ-specific dynamics of human critical illness.

Recent years have seen advances in in vitro platforms—especially 3D cell cultures and microfluidic systems—that recapitulate key aspects of human biology in controlled settings [2]. These human-derived models offer mechanistic insights at cellular and tissue levels that traditional animal studies often cannot capture. This raises a pivotal question: can integrating such advanced in vitro models with conventional animal models yield more predictive and translatable findings in critical care research? In this hypothesis article, we posit that a synergistic integration of human-relevant in vitro systems with animal models will improve the fidelity of preclinical research and bridge the bench-to-bedside gap. Specifically, we hypothesize that combining advanced in vitro models derived from human cells—particularly those from ICU patients—prior to in vivo animal studies can unveil human-specific disease mechanisms and therapeutic responses that neither model alone can discern, ultimately improving the clinical translation of critical care research. Below, we outline the rationale for this hypothesis, examine supporting evidence, and discuss how it could be tested and implemented to advance the field.

## Advanced in vitro microfluidic models: new tools for translational critical illness research?

Organ-on-a-chip and body-on-a-chip models are microfluidic cell culture systems that emulate the structural, functional, and mechanical microenvironment of human tissues and offer several key advantages for translational research [3]. These platforms typically consist of perfusable microchannels lined with living human cells, arranged to mimic physiological interfaces, such as the alveolar–capillary barrier or gut–liver axis. By integrating fluid flow, shear stress, and 3D architecture, they reproduce key aspects of organ-level physiology and pathology in vitro, allowing real-time analysis of cellular responses, tissue–tissue communication, and systemic effects in a highly controlled and human-relevant setting. By using primary or iPSC-derived human cells, they avoid the interspecies differences that limit animal-to-human translation.

**Fig. 1:**
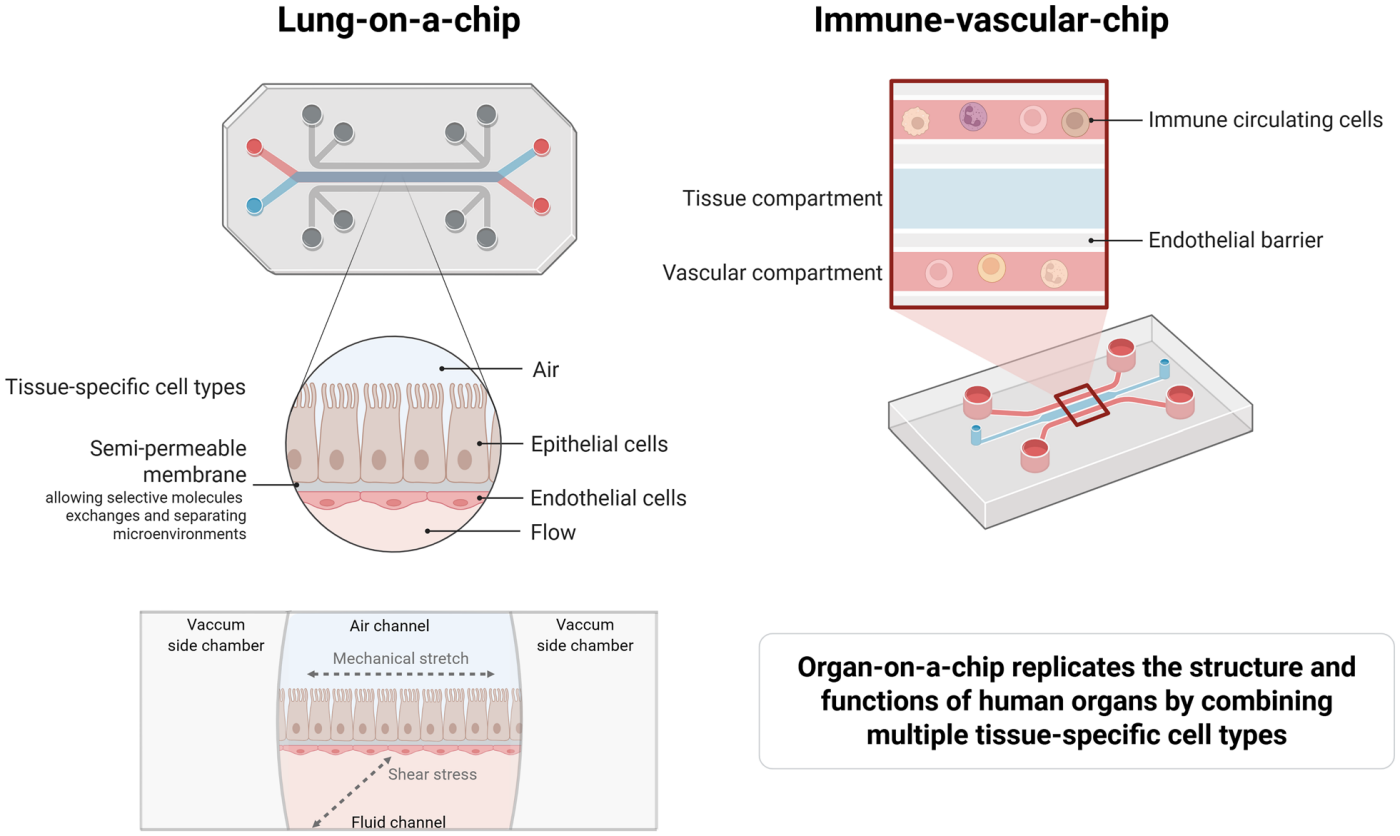
Representative organ-on-a-chip platforms modeling lung and immune–vascular interactions. Left: the lung-on-a-chip mimics the alveolar–capillary interface using epithelial and endothelial cells separated by a semi-permeable membrane, exposed to air, fluid flow, and cycling mechanical stretch to simulate breathing dynamics. Right: the immune–vascular chip features a vascular compartment lined with endothelial cells and perfused with circulating immune cells, enabling the study of immune–endothelial interactions under flow. These platforms replicate key structural and functional features of human organs by combining multiple tissue-specific cell types

*Modeling human disease.* A growing body of evidence supports the potential of these systems to model human pathophysiological processes with high fidelity. For instance, lung-on-a-chip models replicate alveolar–capillary interface dynamics using human epithelial and endothelial cells on a microfluidic platform (Fig. 1). These chips allow real-time visualization of immune cell adhesion, barrier disruption, and cytokine signaling under mechanical stretch—mimicking not just the structural but also the biomechanical environment of human lungs during ventilator-induced lung injury [4]. In a landmark study, Huh et al. showed that IL-2–induced pulmonary edema was exacerbated by cyclic mechanical strain in a lung-on-chip device, key insight into ventilator-associated lung injury that was previously unrecognized in animal models [5]. Building on this concept, Bai et al. reveal how dynamic mechanical stretch modulates the viral infectivity through a mechano-immunological control, offering novel insights into Influenza virus-induced lung injury and host–pathogen interactions [6]. In future applications, co-culturing with macrophages in a lung-on-chip model may more accurately recapitulate coordinated immune responses to an insult, thereby strengthening translational value.

**Fig. 2:**
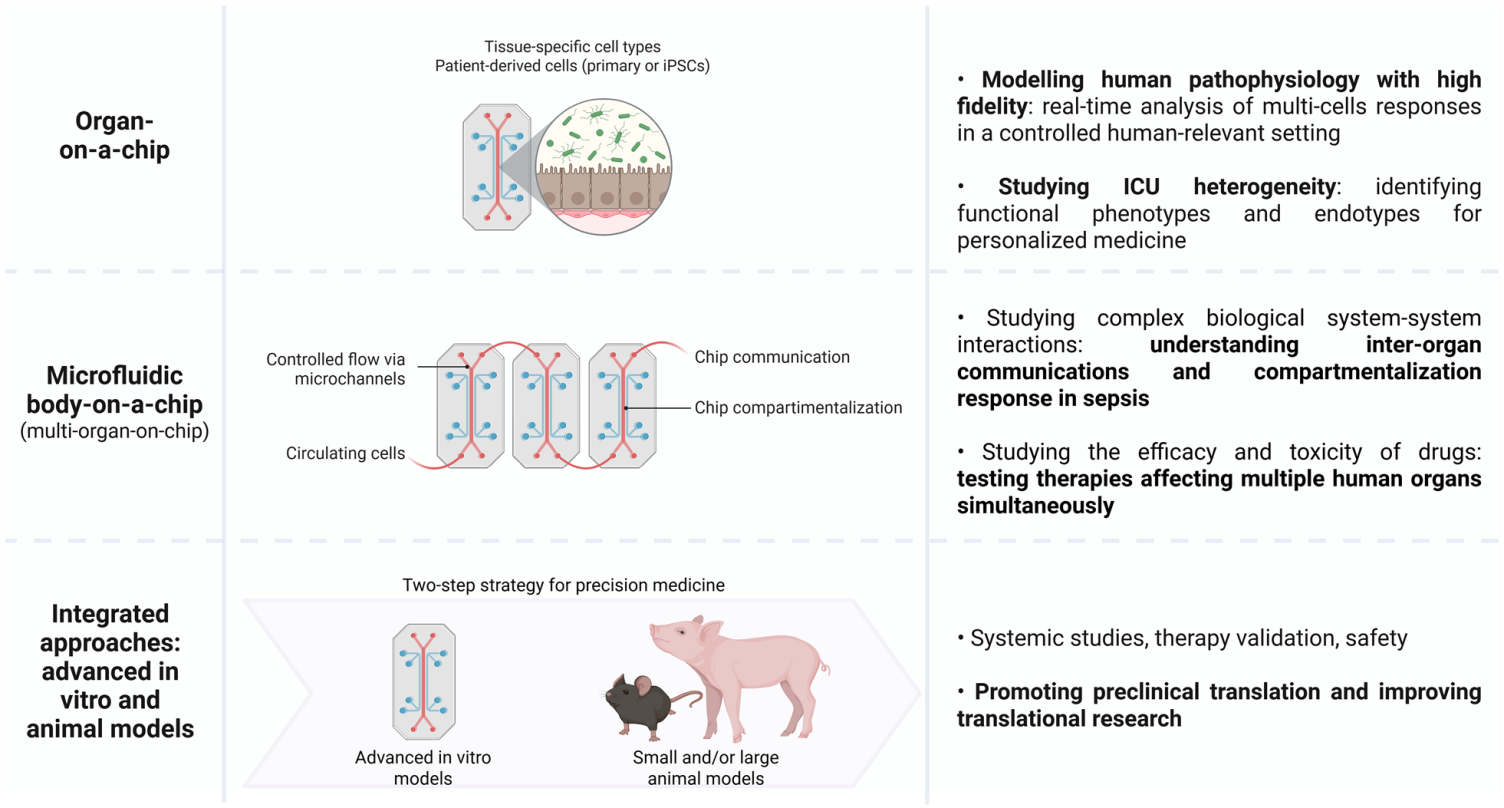
Complementary roles of organ-on-a-chip, body-on-a-chip, and animal models in critical illness research. Integrated approaches combining advanced in vitro systems with animal models support a two-step strategy for precision medicine, linking mechanistic insights with systemic validation to enhance translational outcomes in critical care

*Understanding organ crosstalk.* Similarly, body-on-a-chip systems, comprising interconnected microfluidic organ units, enable the simulation of multi-organ interactions (Fig. 2) and the dissection of organ crosstalk [7]. They allow the study of complex biological interactions across organ systems, providing valuable insight into inter-organ communication and compartmentalized inflammatory responses during conditions such as sepsis. In parallel, they offer a powerful tool for evaluating both the efficacy and toxicity of therapeutic compounds by testing their effects on multiple human organs simultaneously [8]. These platforms have successfully recreated key features of sepsis pathophysiology, such as leukocyte trafficking, endothelial activation, and pathogen invasion, under controlled flow conditions, in a way not possible in animal models [9].

*Identification of functional phenotypes (endotypes).* Such systems offer the ability to identify functional cellular phenotypes. Yang et al. used an organ-on-chip platform with a human lung microvascular endothelium under flow, perfused with patient-derived immune cells, to analyze neutrophil behavior in ICU sepsis patients (Fig. 1). The authors identified three distinct neutrophil phenotypes—hyperimmune, hypoimmune, and hybrid—based on ex vivo neutrophil adhesion and transmigration patterns across the endothelium. These functional phenotypes correlated with clinical severity. This immune–vascular chip illustrates how multi-cell, two-compartment microsystems can capture patient-specific immune dysregulation and endothelial interactions in sepsis, enabling stratification of sepsis endotypes for personalized therapy [10].

*Patient heterogeneity and personalized medicine.* Critical illnesses are notorious for their patient-to-patient heterogeneity—co-morbidities, age, baseline immune status, and other factors. Traditional animal models, usually using young, healthy inbred animals, cannot fully replicate this variability. Advanced in vitro platforms open new frontiers in precision medicine. First, by relying on human cells, these systems overcome the interspecies barrier that limits the translational value of animal studies.

Second, by incorporating cells directly derived from ICU patients, they can mirror individual-specific genetic, immunological, and metabolic characteristics, thereby bringing the complexity of the ICU patient into the laboratory. This provides a unique opportunity to analyze inter-individual variability in disease trajectories, drug efficacy, and toxicity [11]. Third, by identifying endotypes, these models offer the possibility to stratify critically ill patients into biologically defined subgroups. In critical illness, where therapies often fail due to “one-size-fits-all” assumptions [12], personalized organ-on-chip models may help predict who will respond to targeted interventions.

## Implementing an integrated approach

**Fig. 3:**
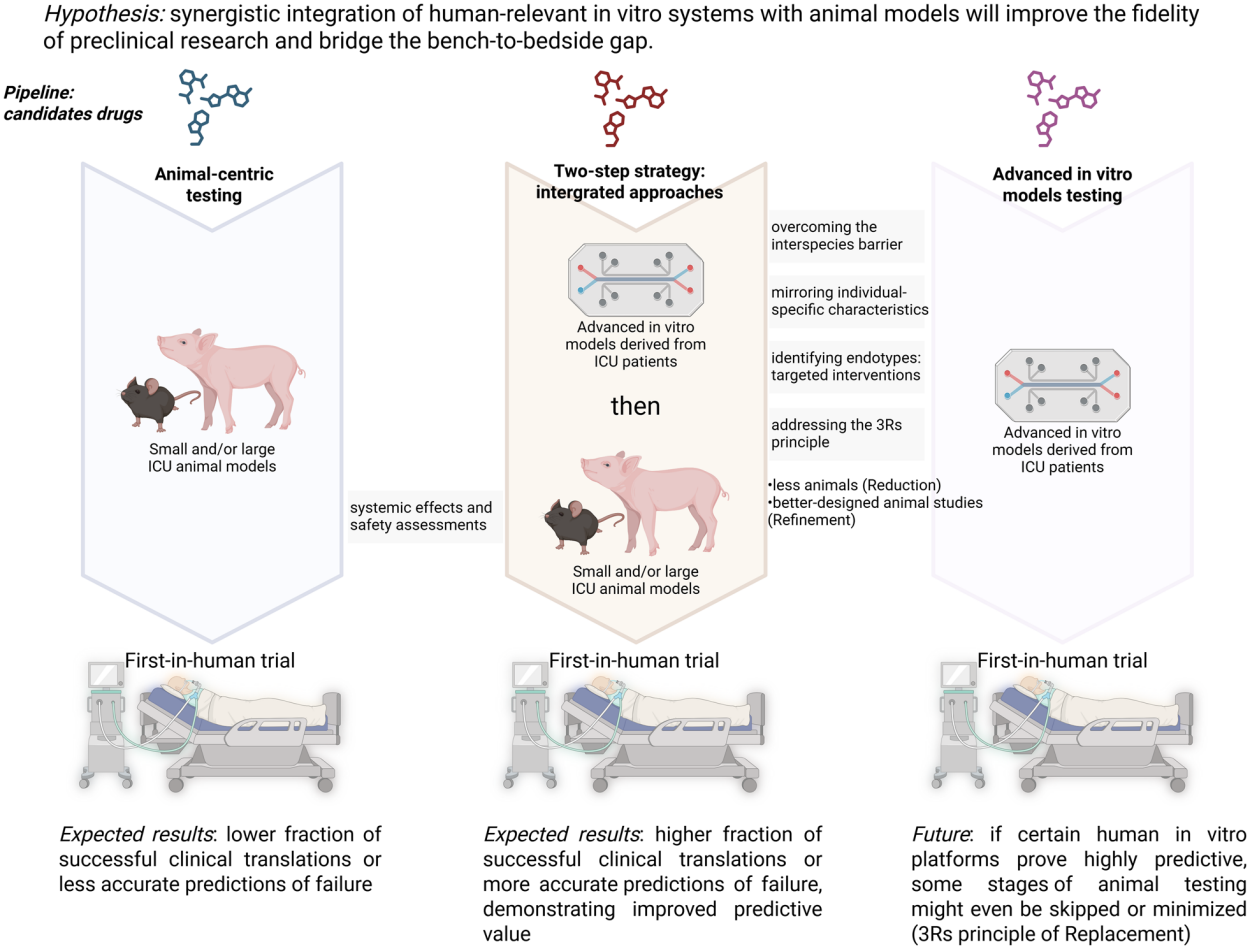
Conceptual framework for integrating advanced in vitro models prior to animal studies in critical illness research. Compared to animal-centric testing alone, a two-step integrated strategy, using advanced in vitro models derived from human cells—particularly those from ICU patients—prior to in vivo animal studies, may improve predictive value, thereby increasing the likelihood of successful clinical translation

While evidence outlined above for the value of advanced in vitro platforms continues to emerge, achieving the full potential of an integrated in vitro–in vivo research pipeline will require overcoming practical challenges and adapting current research paradigms (Fig. 3).

*Addressing the 3Rs principle.* First, it is important to acknowledge that animal models remain indispensable, particularly for validating whole-organ and systemic effects and for safety assessments prior to first-in-human trials. Our hypothesis suggests a strategic two-step approach: use in vitro models to gain mechanistic and human-specific insights, then study those findings in animal studies before moving to patients. For example, promising drug candidates or therapeutic targets might be first screened on human organ chips for efficacy and off-target effects on multiple cell types; only the most promising and safe candidates would then advance to animal testing, thereby reducing the number of animals needed (3Rs principle of Reduction) and focusing in vivo experiments on those with a higher likelihood of success. Such refinement of animal use (3Rs principle of Refinement) means animal studies can be better designed and more informative, guided by hypotheses generated from human-cell data. In the long run, if certain human in vitro platforms prove highly predictive, some stages of animal testing might even be skipped or minimized (3Rs goal of Replacement), though this will require convincing validation and likely regulatory changes.

*Barriers to implementation.* Technical issues such as scalability, reproducibility, and the incorporation of additional physiological features (e.g., neural and hormonal feedback loops, or multiple immune cell types in co-culture) are areas of development. For instance, achieving robust co-culture in a lung-on-chip is technically challenging but necessary to fully recapitulate the lung’s response to injury or infection. Investment in engineering more user-friendly and standardized organ-on-chip devices will be needed for them to become routine research tools. Expertise and training present another hurdle: researchers will need interdisciplinary skills in cell biology, bioengineering, and computational analysis to use these systems effectively. Additionally, there may be resource and funding constraints, as these sophisticated models can require specialized equipment and materials. Funding agencies and industry partners will have to prioritize such integrative research for it to flourish.

*Regulatory agencies.* On a broader scale, regulatory acceptance of organ-on-chip data is still in its infancy. Regulatory acceptance of organ-on-chip data is growing. In the United States, for example, the FDA Modernization Act 2.0 (2022) now permits developers to use non-traditional preclinical methods—including microphysiological systems like organ chips—to help satisfy safety and efficacy requirements [13]. The FDA has launched pilot programs to qualify these novel platforms, signaling a growing willingness to consider in vitro results in regulatory submissions.

## How the hypothesis could be tested?

Our hypothesis that integrating in vitro and in vivo models improves translation is testable (Fig. 3). One way to validate it would be through retrospective analyses and prospective studies [1]. Retrospectively, one could examine past drug development programs: where there failed clinical therapies that an organ-on-chip model might have flagged as ineffective or toxic due to human-specific responses? Conversely, can we identify treatments that were abandoned because they failed in animal trials but might have succeeded in humans? Those could be re-tested on modern human-organ models to see if negative or positive effects are evident [2]. Prospectively, an integrated pipeline could be implemented for a set of candidate drugs in an area with high animal-to-human failure rates (such as sepsis or ARDS). Half of the candidates would go through traditional animal-centric testing, while the other half are prioritized using organ-on-chip screening before animal testing. If the hypothesis is correct, the latter approach should yield a higher fraction of successful clinical translations or more accurate predictions of failure, demonstrating improved predictive value.

Additionally, mechanistic hypotheses arising from chips can be tested in animal models and early-phase clinical trials. For example, if a sepsis-on-chip model identifies an immune phenotype that predicts non-response to a therapy, one could test in an animal model genetically engineered to mimic that phenotype or stratify patients in a trial by that biomarker to see if outcomes differ. Success in these experiments would support the value of the integrated approach.

## Conclusion

In summary, we hypothesized that an integration of advanced in vitro models derived from ICU patients with animal models will accelerate translational progress in critical illness research. By leveraging the complementary strengths of each approach, researchers may overcome limitations that have historically impeded the development of effective therapies for conditions like sepsis and ARDS. Crucially, this vision is actionable and will benefit from further research. We encourage investigations that directly compare outcomes of an integrated in vitro–in vivo pipeline versus conventional approaches, to formally test the hypothesis. Success would be measured in terms of better alignment with clinical trial results. Ultimately, the synergy of cutting-edge in vitro systems with animal models holds the promise of closing the translational gap—translating bench discoveries to bedside therapies more reliably—and thereby improving outcomes for critically ill patients.

## Data Availability

Not applicable.
